# BDNF/TrkB signaling mediates the anorectic action of estradiol in the nucleus tractus solitarius

**DOI:** 10.18632/oncotarget.21062

**Published:** 2017-09-19

**Authors:** Ling Shen, David Q.H. Wang, Meifeng Xu, Stephen C. Woods, Min Liu

**Affiliations:** ^1^ Department of Pathology and Laboratory Medicine, University of Cincinnati College of Medicine, Cincinnati, OH, USA; ^2^ Department of Psychiatry and Behavioral Neuroscience, University of Cincinnati College of Medicine, Cincinnati, OH, USA; ^3^ Department of Medicine, Division of Gastroenterology and Liver Diseases, Marion Bessin Liver Research Center, Albert Einstein College of Medicine, Bronx, NY, USA

**Keywords:** BDNF, TrkB receptor, estrogen, food intake, obesity

## Abstract

Although compelling evidence indicates that estradiol (E2) acts in the nucleus tractus solitarius (NTS) to reduce food intake, the underlying mechanisms are largely unknown. We now report that estrogen's anorectic action occurs through enhancing the strength of brain-derived neurotrophic factor (BDNF) and tropomyosin receptor kinase (TrkB) signaling in the NTS. Intra-4th-ventricular administration of a low dose of BDNF reduced food intake to a greater extent in ovariectomized (OVX) rats cyclically treated with E2 than in vehicle-treated OVX rats, implying that cyclic E2 replacement increases BDNF's satiating potency. OVX significantly decreased *bdnf* gene expression in the NTS, and this was reversed by cyclic replacement of E2. Treatment of cultured primary neuronal cells from embryonic rat brainstem with E2 or PPT (ERα agonist), but not with DPN (ERβ agonist), significantly increased *bdnf* mRNA levels, indicating that ERα is the primary receptor mediating E2's stimulatory effect on *bdnf* gene expression. Administration of the selective TrkB antagonist, ANA-12, directly into the NTS significantly attenuated E2-induced reductions of food intake and body weight gain in OVX rats, indicating that TrkB receptor activation is necessary for E2's anorectic effect. Finally, relative to controls, OVX mice with *bdnf* gene knockdown specifically in the NTS had a blunted feeding response to E2. These data collectively imply that BDNF/TrkB receptor signaling in the NTS is a downstream mediator of E2 in the control of energy intake.

## INTRODUCTION

Estrogens, especially estradiol (E2), have potent effects on energy intake [1, 2]. In female mammals, including rodents and humans, eating decreases during the peri-ovulatory period of the ovarian cycle due to the preceding and coincident increases in circulating estrogen [3]. Disruption of rat ovarian cycling by ovariectomy (OVX) significantly increases daily food intake and promotes body weight gain, and these can be normalized by cyclic E2 replacement that mimics a normal ovarian cycle [4]. Despite these well-known anorectic effects of E2, the mechanisms underlying E2's catabolic actions are largely unknown.

Considerable evidence suggests that E2 exerts its catabolic action indirectly through enhancing the strength of other signals implicated in the direct control of food intake and energy expenditure [5]. Brain-derived neurotrophic factor (BDNF) and its receptor, Tropomyosin receptor kinase B (TrkB), is a strong candidate in this regard. Previous reports indicate that BDNF and TrkB signaling is critical in the central regulation of energy homeostasis [6, 7]. In humans, mutations in either the *bdnf* or the *TrkB* genes are associated with obesity accompanied by hyperphagia [8, 9]. Central administration of BDNF reduces food intake and increases energy expenditure in rodents [10–12]. While global knockout of the *bdnf* gene is lethal [13], mice heterozygous for BDNF display chronic hyperphagia and age-dependent obesity [14, 15], and selective deletion of *bdnf* in the brain causes a phenotype characterized by hyperphagia and obesity, as well as increased abdominal white adipose tissue, changes that are significantly more pronounced in females [16]. Similarly, mice with central *TrkB* gene deletion also have increased body weight, an effect that is significantly more robust in females than in males [17]. These findings highlight the need for focused examination of the role of BDNF/TrkB receptor signaling in mediating E2's anorectic effects.

The goal of the present studies was to determine whether E2 inhibits food intake through enhancing BDNF/TrkB signaling in the nucleus tractus solitarius (NTS) of female rats and mice. We focused on the NTS, a key brain stem nucleus for food intake regulation, as a target area because previous studies have demonstrated that the NTS is critical to E2's anorectic action [18] and that BDNF acts in this area to influence food intake and body weight [12]. We therefore determined the effects of physiologic-dose cyclic E2 treatment on 1) sensitivity to the anorexic effect of intra-4th-ventricularly administered BDNF in OVX rats, and 2) the expression of the *bdnf* gene in the NTS. Cultured primary neuronal cells from embryonic brainstems of rats were used to determine which estrogen receptor potentially mediates E2's stimulation on *bdnf* gene expression. Additionally, we assessed whether NTS delivery of the TrkB selective antagonist, ANA-12, or else *bdnf* gene knockdown specifically in the NTS, attenuates E2's anorectic action. Overall, these experiments were designed to determine whether the BDNF/TrkB signaling in the NTS is necessary and sufficient to mediate the effect of E2 on food intake.

## RESULTS

### The anorectic action of BDNF is enhanced by cyclic E2 replacement in OVX rats

Adult female rats with intra-fourth ventricular cannulas received bilateral ovariectomy (OVX) through a midline abdominal incision, as we reported previously [19]. Five days later, the OVX rats were divided into two weight-matched groups, one being injected subcutaneously (sc) every 4th day with E2 (2 μg) in 100 μl sesame oil for 7 cycles (total 28 days), and the other being injected sc with vehicle (100 μl sesame oil) for the same period.

**Figure 1:**
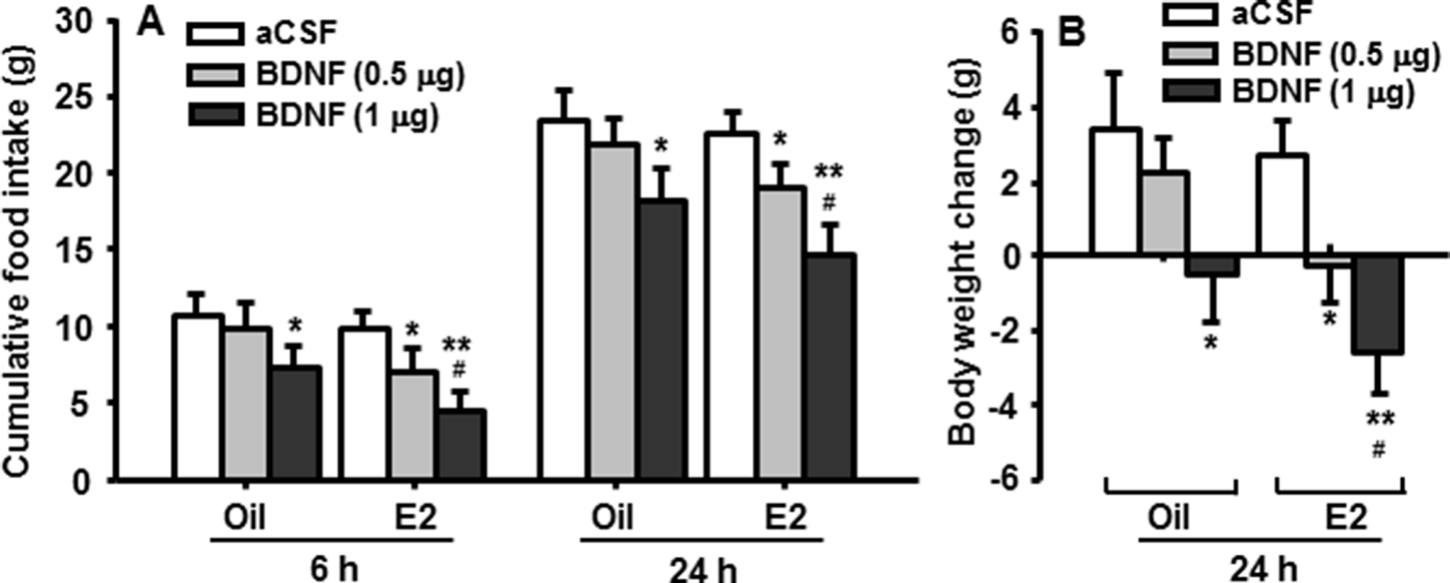
Cyclic E2 replacement enhances the anorectic effect of BDNF in OVX rats (**A**) Food intake was measured at 6 h and 24 h, and (**B**) body weight was measured at 24 h after i4vt administration of BDNF (0.5 or 1 μg). Data expressed as the mean ± SEM, *n* = 7–8 rats per group. *P < 0.05; ^**^P < 0.01, compared with aCSF controls; ^#^*P* < 0.05, compared with the OVX rats receiving 0.5 μg of BDNF.

Both groups of OVX rats, after 4-h fasting, received intra-4th ventricular (i4vt) BDNF (0.5 or 1 μg) or vehicle (aCSF) at the onset of dark, which was 30 h after the most recent sc injection of E2 or oil. We selected these doses of BDNF because they were subthreshold (0.5 μg) and minimally effective (1.0 μg) in male rats, as reported previously [12], and confirmed in a pilot study. Food intake was measured at 6 and 24 h after BDNF administration. I4vt BDNF (0.5 μg) significantly decreased food intake (Figure 1A) and body weight gain (Figure 1B) in E2-treated OVX rats, but not in oil-treated OVX rats. No significant difference in food intake was found at 2, 4 or 36 h after the BDNF administration. The larger dose of BDNF (1 μg) decreased food intake and body weight gain in both groups with the reduction being significantly less in oil-treated rats than in E2-treated rats (Figure 1A and 1B).

### Bdnf gene expression in the NTS of female rats varies across the ovarian cycle

Age-matched (10-wk old) male and female rats were used. The males, which had stable food intake and body weight, were used as a control. Phase of the ovarian cycle was determined by examination of vaginal smears taken daily 4 h prior to dark onset, and estrous was characterized by large clumps of non-nucleated squamous cornified cells, as described previously [20]. The rats were sacrificed at the beginning of the dark (female rats at diestrous and estrous phases of estrous cycle, respectively). The NTS was micropunched and *bdnf* mRNA levels were measured with quantitative real-time PCR (qPCR) [21].

**Figure 2:**
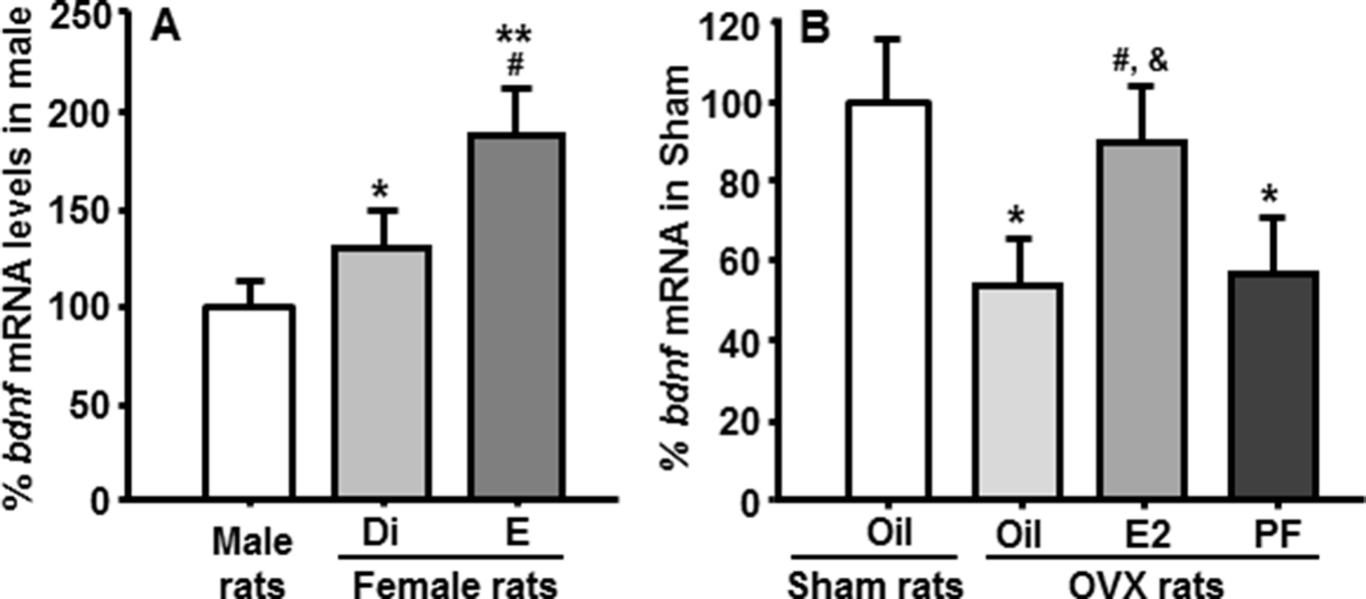
Comparison of *bdnf* mRNA levels in the NTS measured by qPCR (**A**) *Bdnf* mRNA levels in the NTS of female rats were significantly higher than those of male rats, and *bdnf* gene expression in female rats at estrus was significantly higher than those of rats at diestrus. Mean ± SEM, *n* = 7–8. *P < 0.05 and ^**^P < 0 .01, compared with male rats; ^#^*P* < 0.05, compared to rats at diestrus. Di, diestrus phase; E, estrous phase. (**B**) The decreased *bdnf* mRNA levels induced by OVX were restored by E2 replacement, but not by pair-feeding. The pair-fed (PF) rats were provided with the same amount of food consumed by E2-treated OVX (OVX + E2) rats each day. *P < 0.05, compared with sham oil-treated rats; ^#^*P* < 0.05, compared with OVX oil-treated rats; ^&^*P* < 0.05, compared with OVX + pair-fed rats.

As depicted in Figure 2A, *bdnf* mRNA levels in the NTS of female rats were significantly higher than those in male rats at both diestrous (Di) and estrous (E), and they were significantly higher at estrous than at diestrus, consistent with the preceding increase in circulating E2 levels during the estrus phase [4].

### Cyclic E2 replacement stimulates bdnf gene expression in the NTS of OVX rats

Female rats received OVX or sham surgery, as we reported previously [19]. The sham-operation procedure consisted of exposure of the ovaries on both sides, but leaving them intact. Half the OVX rats then received cyclic E2 (2 μg) replacement and the other half received vehicle (oil) for 7 cycles. One group of OVX-vehicle rats was pair-fed by being provided with the same amount of food consumed by E2-treated OVX rats each day. The rats in the sham group were allowed to eat freely and were treated with oil. The OVX rats were sacrificed at the onset of dark on the 2nd day after the last E2 or vehicle injection and then the NTS was micropunched [20]. The sham-operated rats were sacrificed at the onset of dark when they were in the estrous phase.

OVX rats treated with oil had significantly reduced *bdnf* mRNA in the NTS, compared with sham-operated females. This reduction was almost completely restored by cyclic replacement with E2, but not by pair-feeding, implying that E2 directly regulates *bdnf* gene expression in the NTS, and that this is not secondary to the changes in energy intake (Figure 2B).

### An ERα agonist stimulates bdnf gene expression in cultured neuronal cells

**Figure 3:**
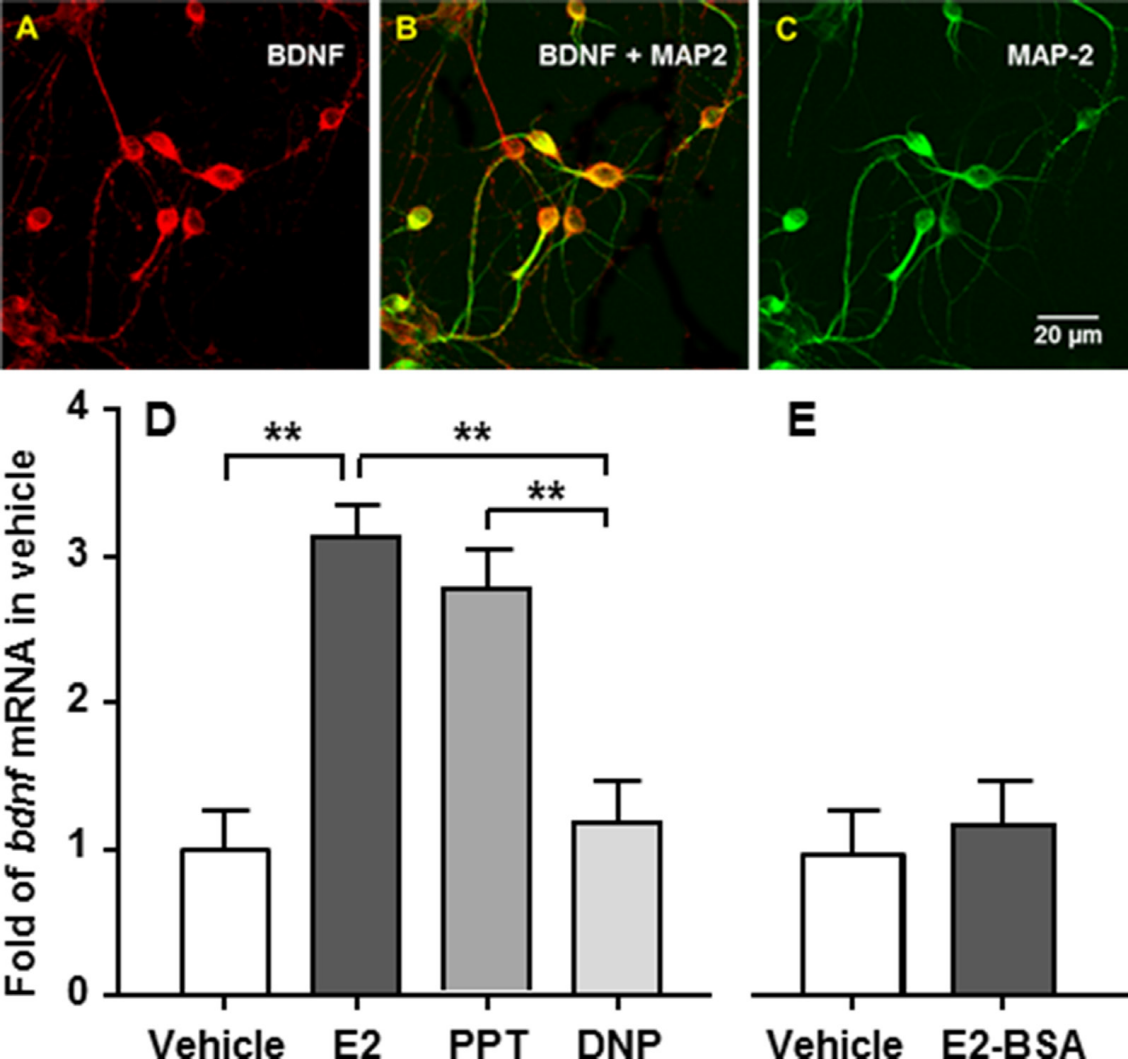
*Bdnf* gene expression is stimulated by E2 or an ERa, but not an ERb, agonist in cultured embryonic brainstem cells (**A–C**) The cells were stained with antibodies against BDNF (*red*) and MAP-2 (*green*). The co-localization of BDNF with MAP-2, a neuronal marker, is reflected as an orange color. (**D**) The cells were treated for 24 h with 10 nM E2, PPT, or DPN, respectively. Both E2 and PPT, but not DPN, significantly stimulated *bdnf* gene expression, compared with vehicle controls. (**E**) To determine whether a nongenomic mechanism is involved, the cultured neuronal cells were treated with *E2-BSA* or vehicle. No significant difference was found between *E2-BSA* and vehicle-treated cells. Mean ± SE; *n* = 3. ^**^P < 0.01.

To determine which type of estrogen receptor mediates E2's stimulatory action on *bdnf* gene expression, primary neuronal cells were prepared from Long-Evans rat embryos on the 18th day of gestation as we reported previously [20]. After incubation for 3 days, half of the medium was replaced with fresh medium containing cytosine arabinofuranoside (10 μM; Sigma), an anti-mitotic agent, in order to reduce the population of non-neuronal cells [22], and additional incubation at 37°C was carried out for an additional 3 days. Six days after seeding, using dual-labeling immunocytochemistry, BDNF was found to be present in cultured primary embryonic brainstem neurons and colocalized with MAP2, a neuronal marker (Figure 3A–3C).

The cultured primary neuronal cells were then treated with 10 nM E2, PPT (ERα agonist), or DPN (ERβ agonist), for 24 h, as described previously [20]. Treatment with 10 nM E2 or PPT, but not with DPN, significantly stimulated *bdnf* gene expression, compared with vehicle controls. *Bdnf* mRNA levels in E2-treated cells were comparable with those in PPT-treated cells (Figure 3D).

To determine whether a nongenomic mechanism is involved in E2's effect on *bdnf* gene expression, we treated cultured neuronal cells with E2 linked to bovine serum albumin (*E2-BSA*) for 24 h, an estrogen formulation that can activate membrane-associated ERs but cannot enter cells due to its large size and charge properties [23]. No significant difference in *bdnf* mRNA levels was found between E2-BSA- and vehicle-treated cells (Figure 3E), implying that the nuclear ERα is the primary mediator of E2's action on *bdnf* gene expression.

### Antagonism of TrkB receptor signaling in the NTS diminishes E2's anorectic action

BDNF acts through its receptor, TrkB, to regulate energy balance [24]. To determine whether NTS TrkB mediates the inhibitory effect of E2 on food intake, a TrkB antagonist, ANA-12 (13), was directly administered into the NTS. OVX rats with NTS cannulas received E2 (4 μg, sc) or vehicle (oil, 100 μl) at 1000 h [25]. At dark onset on the 2nd day after E2/oil injection, ANA-12 (3 μg, an effective dose at blocking BDNF's anorectic effect [12]) or vehicle (DMSO, 100 nl) was injected directly into the NTS. The four counterbalanced conditions were as follows: control (oil followed by DMSO), ANA-12 (oil followed by ANA-12), E2 (E2 followed DMSO), and the combination (E2 followed by ANA-12). Food intake and body weight were measured 24 h after the injection of ANA-12/DMSO.

**Figure 4:**
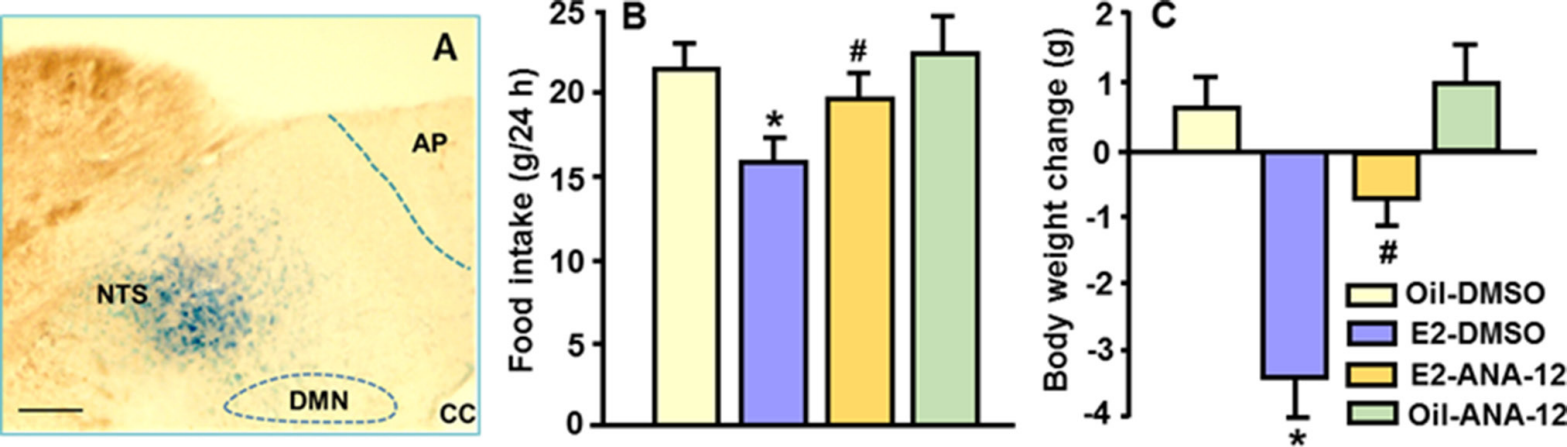
Antagonism of TrkB receptor signaling in the NTS attenuated E2's anorectic action The highly TrkB-selective receptor antagonist, ANA-12, was used to determine whether the NTS TrkB receptor mediates the reduction of food intake induced by acute treatment of E2. (**A**) Photomicrograph illustrating the injection site (NTS), as identified by 0.2 μl of methylene blue. AP, area postrema; cc, central canal; DMN, dorsal motor nucleus of the vagus. Scale 100 μm. (**B** and **C**) E2 significantly reduced food intake and body weight gain, and these effects were significantly attenuated by pre-administration of a dose of 3 μg ANA-12. Mean ± SEM, *n* = 6–8. *P < 0.05, compared with vehicle-treated rats; ^#^*P* < 0.05, compared with E2-treated rats.

Consistent with a previous report [12], ANA-12 at a dose of 3 μg did not significantly affect food intake or body weight. In OVX rats, E2 significantly reduced food intake and body weight gain at 24 h, and pre-administration of ANA-12 significantly attenuated these suppressive effects; that is, the combination condition differed significantly from the E2 alone condition (Figure 4B and 4C). There was no significant difference in body weight change between the E2-ANA-12 group and the oil-ANA-12 group.

### E2's anorectic effect is attenuated in mice with bdnf gene knockdown specifically in the NTS

If BDNF signaling in the NTS underlies E2-dependent changes in feeding and body weight, genetic knockdown of *bdnf* specifically in the NTS of mice should cause them to be less responsive to E2, compared with their controls. To test this hypothesis, genetic disruption of the *bdnf* gene was achieved by stereotaxic injection of an AAV8-Cre virus [26, 27] directly into the NTS of adult female *bdnf*^flox/flox^ mice, in which exon V of *bdnf* is flanked by loxP sites [28]. AAV8-GFP was used as a negative control. Five days later, the mice were fasted overnight and received bilateral OVX, as described previously [19]. The OVX mice were then divided into 2 weight-matched groups of each viral treatment. One group received E2 (2 μg, sc) in oil every 4th day for 7 cycles and the other group received vehicle (100 μl sesame oil, sc) for the same period. Food intake and body weight were monitored daily and body fat was assessed by nuclear magnetic resonance (NMR) before the end of experiment.

**Figure 5:**
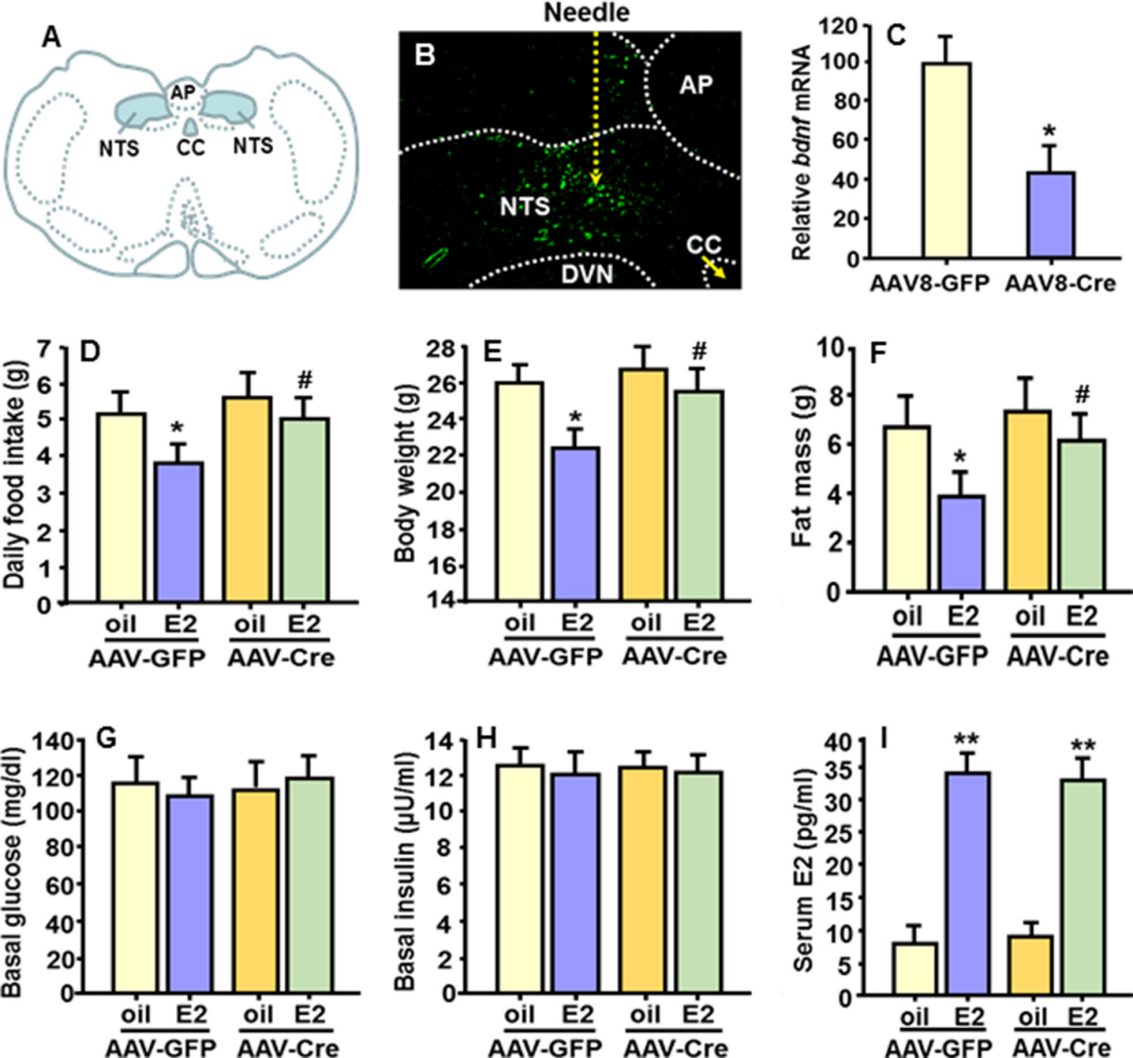
*Bdnf* gene knockdown specifically in the NTS diminished E2's anorectic effect (**A**) Schematic drawing depicting the location of the NTS of mouse brain. (**B**) Representative micrograph showing expression of green fluorescent protein (GFP) of AAV8 virus in the NTS of *bdnf*^flox/flox^ mice as determined under fluorescence microscopy. (**C**) qPCR evaluation of *bdnf* mRNA levels in the NTS punches of AAV-Cre- and AAV-GFP-injected *bdnf*^flox/flox^ mice. Mean ± S.E., *n* = 15–16 per group. *P < 0.05, compared with the OVX mice treated with AAV-GFP virus. (**D**–**F**) Comparison of food intake, body weight and body fat mass among OVX mice with *bdnf* gene knockdown specifically in the NTS and OVX control mice, both receiving cyclic treatment of E2 or oil. (**G**–**I**) Comparison of blood glucose, plasma insulin and E2 levels among the 4 groups. Mean ± S.E., *n* = 7–8. *P < 0.05; ^**^P < 0.01, compared with the OVX mice treated with oil; ^#^*P* < 0.05, compared with OVX AAV8-GFP-control mice treated with E2.

On the 2nd day after the last E2/oil treatment, 4 h-fasted mice were decapitated and brains were quickly dissected and rinsed in ice-cold saline. The brainstem area containing the NTS, from approximately –7.2 to –8.0 mm posterior to bregma [29], was sectioned at consecutive intervals of 15 μm (for examining GFP expression in the NTS) and then 200 μm (for the NTS micropunch). All micropunches were snap-frozen in dry ice, immediately prepared for RNA extraction, and *bdnf* mRNA levels were measured by qPCR as described previously [21]. Animals with correct GFP location (Figure 5A) and successful *bdnf* gene knockdown were included in the data analyses.

Figure 5B is a representative micrograph documenting the expression of green fluorescent protein (GFP) of the AAV8 virus in the NTS as determined under fluorescence microscopy. By the end of experiment, 6 wk after the injection, *bdnf* mRNA levels were significantly reduced (average 58% reduction) in NTS punches of AAV8-Cre-injected *bdnf*^flox/flox^ mice, relative to controls (AAV8-GFP-injected *bdnf*^flox/flox^ mice) (Figure 5C).

Consistent with what we found in OVX rats, in OVX AAV8-GFP-control mice, E2 replacement significantly reduced food intake and body weight gain relative to oil-treatment (Figure 5D and 5E). However, these effects were greatly attenuated in OVX *bdnf gene* knockdown (AAV8-Cre-treatet) mice; i.e., the *bdnf gene* knockdown mice ate significantly more food and gained more body weight than E2-treated OVX AAV8-GFP-control mice. There was no significant difference in food intake or body weight between the E2-treated and the oil-treated OVX *bdnf gene* knockdown mice (Figure 5D and 5E). Consistent with the changes in body weight, E2-treated OVX AAV8-GFP-control mice displayed significantly reduced body fat mass, compared with oil controls, and this effect was diminished in OVX *bdnf gene* knockdown mice (Figure 5F). No difference in lean mass was found (data not depicted). Additionally, there were no differences in blood glucose or plasma insulin levels among all groups (Figure 5G and 5H). As expected, the OVX mice had very low plasma E2 levels, and this was significantly increased in OVX+E2 mice (Figure 5I), and was within the physiological range [30].

## DISCUSSION

Compelling evidence suggests that E2 exerts its catabolic action indirectly via enhancing the strength of other signals implicated in the direct control of food intake and energy expenditure. BDNF is a strong candidate in this regard since it potently reduces food intake and increases energy expenditure [31]. Central deletion of *bdnf* or *TrkB* gene causes a metabolic phenotype characterized by hyperphagia and obesity that is significantly more pronounced in females than in males [16, 17].

Although E2 acts within the brain to decrease meal size [5], the specific brain site(s) remains unclear. Previous studies demonstrated that the NTS is important for E2's anorectic action [1], and ERα in the dorsal hindbrain is predominately localized in the NTS [32]. Administration of a low dose of E2 (0.2 μg) onto the surface of the hindbrain over the NTS, but not subcutaneous administration of the same E2 dose, significantly decreased food intake and activated ERα-containing neurons in the NTS without a change in systemic plasma E2 [18]. All of these observations support our decision to focus on the NTS as the location of the interaction of E2 and BDNF/TrkB signaling in the control of food intake.

Importantly, E2 increased the anorectic effect of BDNF in OVX rats; i.e., at a subthreshold dose (0.5 μg, i4vt), BDNF significantly reduced food intake in E2-treated, but not in oil-treated OVX rats. Although a larger dose of BDNF (1 μg, i4vt) decreased food intake and body weight gain in oil-treated OVX rats, these effects were significantly less than what occurred in E2-treated OVX ones (Figure 1). Our findings provide strong evidence that a near-physiological dose of E2 is sufficient to increase BDNF-induced reduction in food intake and body weight. Since the rats received a regimen of E2 treatment that mimics the changes and plasma levels of E2 in normally cycling female rats [4, 19], it is likely that the decrease in food intake during estrous, which is well characterized in intact female rats, is mediated, at least in part, by an increase in BDNF/TrkB signaling in the NTS.

Age-matched females weigh significantly less and consume less daily food than males [20], and female rats with intact ovaries exhibit cyclic changes in feeding across the ovarian cycle, being highest at diestrus, declining during proestrus, and reaching a nadir at estrus right after the E2 surge [20]. To determine whether *bdnf* gene expression within the NTS is modulated under normal physiological conditions, we compared *bdnf* mRNA levels between male and female rats at both diestrous and estrous phases. The E2 levels of diestrous rats are comparable with those of vehicle-treated OVX rats or male rats, and the E2 level of estrous rats is comparable with that of sham rats on the day of estrus and of E2-treated OVX rats on day following a cyclic E2 injection [33]. Notably, *bdnf* gene expression in the NTS of female rats at both diestrous and estrous was significantly higher than in male rats (Figure 2A). More importantly, NTS *bdnf* mRNA levels of rats at estrus were significantly higher than those of female rats at diestrus (Figure 2A), consistent with the preceding increase in circulating E2 [33] and higher ERα gene expression during the estrus phase [34].

In our previous studies [19], we found that OVX induces a significant increase in food intake and body weight, relative to sham-operated intact rats. In contrast, food intake in OVX rats receiving cyclic E2 replacement varies cyclically with frequencies and amplitudes similar to those in rats with intact ovaries. The E2-treated OVX rats slowly gained body weight at a rate, which was close to that of sham-operated rats after 3 cycles of E2 treatment [19]. Oil-treated OVX rats showed significantly reduced *bdnf* mRNA levels in the NTS, compared with sham-operated female rats. This reduction was almost completely restored after cyclic E2 replacement, but not by pair-feeding, implying that E2 directly, and not secondary to changes in energy intake, regulates *bdnf* gene expression in the NTS (Figure 2B). These results also suggest that a physiological dose of E2 acts on a population of cells in the NTS to up-regulate transcription of the gene that encodes BDNF and, consequently, increase the anorectic effect of BDNF.

While several estrogen receptors have been identified, ERα has been largely accepted as a key mediator of E2's effects on energy balance. ERα KO mice are obese, whereas ERβ-deficient mice remain lean [35]. Deletion of ERα in mice blocks the anti-obesity effects of estrogen replacement [36]. Consistent with that, administration of an ERα-selective agonist (PPT), but not an ERβ-selective agonist (DPN), promotes hypophagia in OVX rats [25]. If BDNF is an intermediary for E2's anorectic effect, it is likely that E2 acts through ERα to stimulate *bdnf* gene expression. To test this possibility, we treated cultured primary neuronal cells from embryonic brainstems of rats with 10 nM of E2, PPT (ERα agonist), or DPN (ERβ agonist) for 24 h, respectively. Both E2 and PPT, but not DPN, significantly stimulated *bdnf* gene expression, compared to vehicle. (Figure 3D). To determine whether plasma transmembrane estrogen receptors are involved in E2's effect on *bdnf* gene expression, the cultured neuronal cells were treated with E2-BSA, which can activate membrane-associated ERs, but cannot enter cells due to its large size and charge properties [23]. No significant difference was found between E2-BSA and vehicle-treated cells (Figure 3E). Collectively, these data indicate that nuclear ERa is the primary mediator of E2's action on *bdnf* gene expression.

BDNF acts through its receptor, TrkB, to regulate energy balance [24]. To assess whether BDNF/TrkB receptor signaling acts at the downstream of E2 in the control of energy intake, a highly TrkB-selective receptor antagonist, ANA-12, was administered into the NTS of OVX rats with/without E2 replacement. While ANA-12 (3 μg) alone did not markedly affect food intake or body weight, its induced TrkB receptor antagonism significantly attenuated the suppressive effects on food intake and body weight gain elicited by E2 in OVX rats (Figure 4B and 4C), implying that NTS TrkB receptor activation mediates the anorexigenic effects of E2.

The nature of the mechanism by which antagonism of TrkB receptors diminishes E2's anorectic action in the NTS is unknown. ERα and TrkB receptors in the NTS may engage common intracellular signaling pathways, e.g. phosphatidyl inositol 3-kinase (PI3K) signaling, which has been implicated in the regulation of energy homeostasis [37]. Previous studies have demonstrated that an ERα-PI3K cascade mediates E2's catabolic effects. Specifically, E2 stimulates gene expression of PI3K subunits [38] and increases phosphorylation of Akt, a PI3K downstream event [39], in the hypothalamus. Since BDNF also activates the PI3K pathway via the TrkB receptor [40], and since genetic inhibition of PI3K activity in VMH neurons elicits an obese phenotype only in female mice [39], the PI3K cascade in the NTS may be a common pathway that integrates the E2-ERα and BDNF-TrkB signals, leading to a synchronized control of energy balance.

Given that BDNF signaling underlies E2-dependent changes in feeding and body weight, genetic knockdown of *bdnf* specifically in the NTS of mice (AAV-Cre-mice) should cause them to be less responsive to E2, compared with their controls (AAV-GFP-mice). Consistent with this hypothesis, cyclically E2-treated OVX control mice consumed less food, gained less body weight, and had lower body fat than oil-treated OVX control mice. In contrast, the E2-treated OVX *bdnf* gene-knockdown mice ate more food and gained more body weight with higher body fat than E2-treated OVX control mice (Figure 5D and 5E). No significant differences in food intake, body weight or body composition occurred between the E2-treated and the oil-treated OVX *bdnf* gene knockdown mice, indicating that E2 was significantly less efficacious in these OVX mice. Collectively, these results strongly imply that endogenous NTS BDNF is both necessary and sufficient to mediate the inhibitory effects of E2 on feeding.

In conclusion, the present studies demonstrate that cyclic E2 replacement enhances the satiating potency of BDNF in OVX rats, and also increases the expression of *bdnf* gene in the NTS. In cultured primary neuronal cells from embryonic brainstems of rats, treatment with E2 or PPT (ERα agonist), but not with DPN (ERβ agonist), significantly stimulates *bdnf* gene expression. NTS delivery of the TrkB-selective antagonist, ANA-12, significantly attenuates E2-induced effects on food intake and body weight gain in OVX rats. Moreover, OVX mice with *bdnf* gene knockdown specifically in the NTS have a less response to E2 than OVX control mice. These data collectively suggest that E2's inhibitory action on food intake in normally cycling female rodents is mediated bythe modulation of BDNF signaling in the NTS.

## MATERIALS AND METHODS

### Animals

Adult male and female Long-Evans rats (Harlan, Indianapolis, IN), 9–10 wk old at surgery, were individually housed in a temperature-controlled vivarium on a 12/12-h light/dark cycle. *bdnf*^flox/flox^ mice (Stock No: 004339, Jackson Lab, Bar Harbor, ME) [28] were bred in our lab. Standard rodent diet and water were provided *ad libitum* except where noted. All animal procedures were approved by the Institutional Animal Care and Use Committee of the University of Cincinnati.

### Materials

17β-estradiol-3-benzoate (E2), 17β-E2 linked to bovine serum albumin (E2-BSA) and sesame oil (as vehicle) were purchased from Sigma (St. Louis, MO). 4,4′,4″-(4-propyl-[1H]-pyrazole-1,3,5-triyl) trisphenol (PPT, an ERα agonist) and 2,3-*bis*(4-hydroxyphenyl)-propionitrile (DPN, an ERβ agonist) were from Tocris (Ellisville, MO). Human recombinant BDNF (Chemicon, Temecula, CA) was dissolved in artificial cerebrospinal fluid (aCSF), and the specific TrkB receptor antagonist ANA-12 (R&D systems, Minneapolis, MN) was dissolved in dimethyl sulfoxide (DMSO) [12]. Sheep anti-BDNF antibody (Cat. No: AB1513) was purchased from Millipore (Billerica, MA), and mouse anti-microtubule-associated protein 2 (anti-MAP2) monoclonal antibody (Cat. No: ab11267) was from Abcam (Cambridge, MA). Adeno-associated virus serotype 8 carrying a Cre recombinase transgene (AAV8-Cre, Cat. #: 7062) and control AAV8-GFP virus (Cat. #: 7061) were purchased from Vector BioLabs (Malvern, PA) [26,27]. Cell culture medium was from Invitrogen. All other chemicals were purchased from Sigma.

### I4vt and intraparenchymal (NTS) cannula implantation

Rats were anesthetized with intraperitoneal (ip) injection of ketamine and xylazine and placed in a stereotaxic instrument with lambda and bregma at the same vertical coordinate. As described previously [19], rats received a guide cannula (22-gauge; Plastics One, Roanoke, VA) with its tip positioned 2.0 mm above the 4th ventricle (coordinates: on the midline, 2.5 mm anterior to the occipital suture and 5.4 mm ventral to the skull, with injector aimed 7.4 mm from skull). For the intra-NTS cannulation, a bilateral guide-cannula (22-gauge) was positioned with its tip 2.0 mm above the medial NTS (coordinates: midline ± 0.75, 1.0 mm posterior to the occipital suture and 5.9 mm ventral to the skull, with injector aimed 7.9 mm from skull), as described previously [12]. The cannulas were attached to the skull with dental acrylic and jeweler's screws and then closed with an obturator.

At least 5 days after surgery, cannula placement was verified through measurement of the sympathoadrenal-mediated hyperglycemic response to 5-thio-D-glucose (5TG, 210 μg for the 4th ventricle and 24 μg for the NTS cannulation dissolved in aCSF) [12]. 5TG is a non-metabolizable glucose isomer that causes NTS neurons to initiate counter-regulatory responses to increase plasma glucose levels [41]. Only animals responding with at least a two-fold increase in plasma glucose after 30 min were used for the next procedure [12, 19]. NTS cannula placement was also verified postmortem through verification of the position of 100 nl of methylene blue injection (Figure 4A).

### Ovariectomy and cyclic E2 replacement

Rats with viable i4vt cannulas were fasted overnight, anesthetized and then underwent bilateral OVX through a midline abdominal incision using sterile surgical techniques as described previously [19]. The OVX rats were divided into 2 weight-matched groups beginning 6 days after surgery. Group-1 rats were injected sc every 4th day with E2 (2 μg) in 100 μl sesame oil for 7 cycles (total 28 days), and Group-2 rats were injected sc with vehicle (100 μl sesame oil) for the same period. Injections were given between 0900 and 1000 h. The dose of 2.0 μg E2 was selected because it produced plasma E2 levels similar to peak levels occurring during the ovarian cycle in intact rats and, when administered over an extended period of time, normalized daily food intake and body weight of OVX rats [4, 19].

### qPCR for bdnf mRNA measurement

Total RNA was extracted from the NTS using a PureLink RNA Mini Kit (Ambion-Life Technologies). RNA was quantified using a NanoDrop 2000 (Thermo Scientific). Then, 250 ng of RNA from each sample was used for reverse transcription to cDNA with a Transcriptor First Strand cDNA Synthesis Kit (Roche). *Bdnf* mRNA levels were quantified by quantitative real-time PCR (qPCR) using TaqMan Fast Advanced Master Mix with TaqMan Gene Expression Assay for *bdnf* with a StepOne™ Plus device (Thermo Scientific), and β-actin mRNA levels from each sample were used as internal controls to normalize the mRNA levels, as described previously [21].

### Primary brainstem neuron cultures and treatments

Primary neuronal cells were prepared from Long-Evans rat embryos on the 18th day of gestation as described previously [20]. Brainstems from the fetal brains were dissected out, the tissues were triturated by repeatedly pipetting, and the isolated cells cultured in a serum-free neurobasal medium supplemented with B-27 at a density of 5 × 10^4^ cells/well in 24-well plate. After incubation for 3 days, half of the medium was replaced with fresh medium with cytosine arabinofuranoside (10 μM; Sigma) but without l-glutamic acid, and additional incubation at 37°C was carried out. Six days after seeding, the cultured primary neuronal cells were treated with 10 nM E2, PPT (ERα agonist), and DPN (ERβ agonist) for 24 h, respectively.

### Stereotaxic injections of AAV8-virus into adult mice

Genetic disruption of the *bdnf* gene was achieved by stereotaxic injection of an AAV8-Cre virus into the NTS of adult female *bdnf*^flox/flox^ mice [26, 27]. AAV8-GFP was used as a negative control. The mice were anesthetized and the viral particles (1 × 10^12^ vg/ml) were loaded into a 5-μl Hamilton syringe attached to a *Motorized Integrated Stereotaxic Injector (iSi) system* (*Stoelting Co.*). The viral particles were injected at a volume of 0.25 μl/side over 5 min, with a 10-min pause between sides. The coordinates for mouse NTS are anteroposterior from bregma –7.5 mm, mediolateral ± 0.4 mm, dorsoventral –4.3 mm, based on the atlas of *Paxinos and Watson* [29] and previous reports [42].

### Body fat/lean composition determination

Body fat and lean composition was assessed by nuclear magnetic resonance (NMR) (EchoMRI; EchoMedical Systems, Houston, TX). Unanesthetized mice were placed in a restraint tube and inserted into the nuclear magnetic resonance, providing estimates of total lean tissue, fat tissue, and water [43].

### Measurement of plasma samples

Blood samples were taken from the tail vein, and glucose was assessed with a glucometer (Freestyle; Abbott Diabetes Care, Alameda, CA) [43]. Plasma insulin was measured in the same samples with a rat insulin ELISA kit (Millipore) [43]. Plasma E2 levels were determined by ELISA kits from Cayman Chemical (Ann Arbor, MI, USA).

### Statistics

The data are presented as mean ± standard error (S.E.). Data were analyzed using GraphPad Prism to evaluate normal distribution and variations within and among groups. *In vitro* experiments were carried out in triplicate and performed on three separate occasions. Differences among more than two groups were determined using one-way or two-way ANOVA analyses followed by Student-Newman-Keuls test for comparison between treatments. Student's *t*-test was used for comparison of effects between E2 and vehicle treatments. *P* values less than 0.05 were considered statistically significant.

## Abbreviations

BDNF: brain-derived neurotrophic factor
TrkB receptor: tropomyosin receptor kinase B receptor
NTS: nucleus tractus solitarius
ERα: estrogen receptor-a
i4vt: intra-4th ventricular
OVX: ovariectomy
